# 1-{6-Chloro-2-[(2-chloro-3-quinol­yl)meth­oxy]-4-phenyl-3-quinol­yl}ethan-1-one

**DOI:** 10.1107/S1600536810021203

**Published:** 2010-06-09

**Authors:** F. Nawaz Khan, S. Mohana Roopan, Rajesh Kumar, Venkatesha R. Hathwar, Mehmet Akkurt

**Affiliations:** a Organic and Medicinal Chemistry Research Laboratory, Organic Chemistry Division, School of Advanced Sciences, VIT University, Vellore 632 014, Tamil Nadu, India; b Solid State and Structural Chemistry Unit, Indian Institute of Science, Bangalore 560 012, Karnataka, India; c Department of Physics, Faculty of Arts and Sciences, Erciyes University, 38039 Kayseri, Turkey

## Abstract

In the title compound, C_27_H_18_Cl_2_N_2_O_2_, the 2-chloro­quinoline and 6-chloro­quinoline rings are almost planar, with maximum deviations from their mean planes of 0.072 (1) and 0.044 (1) Å, respectively, for the Cl atoms. The inter­planar angle between these rings is 14.36 (5)°. The inter­planar angle between the 6-chloro­quinoline and phenyl rings is 66.00 (8)°. In the crystal, mol­ecules are inter­linked by weak C—H⋯O, C—H⋯π and π–π stacking [centroid–centroid distances = 3.7453 (10) and 3.7557 (9) Å] inter­actions.

## Related literature

For a related crystal structure containing 2-quinolone, see: Khan *et al.* (2010 ▶). For the biological activity, such as anti­bacterial, anti­cancer, anti­viral and cardiotonic activity of compounds containing 2-quinolone, see: Ukita & Mizuno (1960 ▶); Jayashree *et al.* (2010 ▶); Joseph *et al.* (2002 ▶); Xiao *et al.* (2001 ▶); Roopan & Khan (2009 ▶).
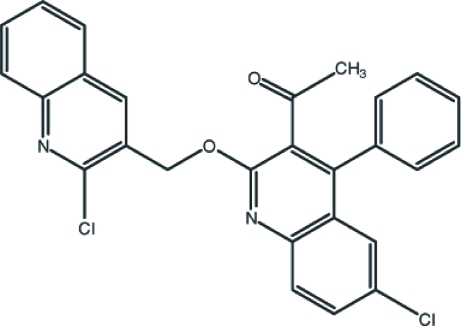


## Experimental

### 

#### Crystal data

C_27_H_18_Cl_2_N_2_O_2_
*M* *_r_* = 473.33
Triclinic, 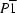

*a* = 9.2694 (3) Å
*b* = 10.8862 (4) Å
*c* = 13.0490 (5) Å
α = 100.615 (3)°
β = 103.570 (3)°
γ = 111.894 (4)°
*V* = 1132.51 (9) Å^3^
*Z* = 2
Mo *K*α radiation
μ = 0.32 mm^−1^
*T* = 295 K
0.25 × 0.21 × 0.14 mm

#### Data collection

Oxford Xcalibur Eos (Nova) CCD detector diffractometer
Absorption correction: multi-scan (*CrysAlis PRO RED*; Oxford Diffraction, 2009 ▶) *T*_min_ = 0.925, *T*_max_ = 0.957
24246 measured reflections
4918 independent reflections
3250 reflections with *I* > 2σ(*I*)
*R*_int_ = 0.037

#### Refinement

*R*[*F* ^2^ > 2σ(*F* ^2^)] = 0.040
*wR*(*F* ^2^) = 0.109
*S* = 1.05
4918 reflections
300 parameters
H-atom parameters constrained
Δρ_max_ = 0.27 e Å^−3^
Δρ_min_ = −0.35 e Å^−3^

### 

Data collection: *CrysAlis PRO CCD* (Oxford Diffraction, 2009 ▶); cell refinement: *CrysAlis PRO CCD*; data reduction: *CrysAlis PRO RED* (Oxford Diffraction, 2009 ▶); program(s) used to solve structure: *SHELXS97* (Sheldrick, 2008 ▶); program(s) used to refine structure: *SHELXL97* (Sheldrick, 2008 ▶); molecular graphics: *ORTEP-3 for Windows* (Farrugia, 1997 ▶); software used to prepare material for publication: *WinGX* (Farrugia, 1999 ▶).

## Supplementary Material

Crystal structure: contains datablocks global, I. DOI: 10.1107/S1600536810021203/fb2195sup1.cif

Structure factors: contains datablocks I. DOI: 10.1107/S1600536810021203/fb2195Isup2.hkl

Additional supplementary materials:  crystallographic information; 3D view; checkCIF report

## Figures and Tables

**Table 1:** Hydrogen-bond geometry (Å, °) *Cg*1 is the centroid of the N1/C1–C3/C8/C9 ring.

*D*—H⋯*A*	*D*—H	H⋯*A*	*D*⋯*A*	*D*—H⋯*A*
C3—H3⋯O1	0.93	2.36	2.703 (2)	101
C6—H6⋯O2^i^	0.93	2.52	3.296 (3)	142
C22—H22⋯*Cg*1^ii^	0.93	2.95	3.683 (3)	137

Symmetry codes: (i) 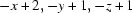; (ii) 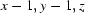.

**Fig. 1.**
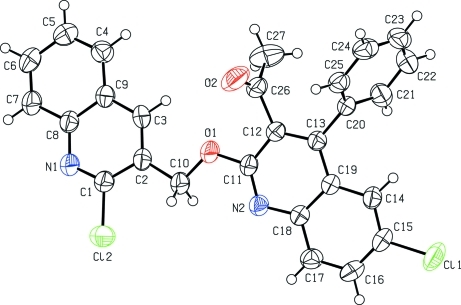
A view of the title molecule, showing the atom-numbering scheme. The displacement ellipsoids are drawn at the 50% probability level.

## supplementary crystallographic information

### Comment

Quinolones have emerged as one of the important classes among chemotherapeutic drugs for treatment of various bacterial infections. The quinolones, precisely the compounds with 2-quinolone moiety, show interesting biologic activities such as antibacterial, anticancer, antiviral and cardiotonic ones (Ukita & Mizuno, 1960; Jayashree *et al.*, 2010; Joseph *et al.*, 2002; Xiao *et al.*, 2001). In continuation of our previous work (Roopan *et al.*, 2009; Khan *et al.*, 2010), we report the structure of a new compound, 3-acetyl-2(2-chloroquinolin-3-yl)methoxy-6-chloro-4-phenylquinoline.

In the title molecule, as shown in Fig. 1, the 2-chloroquinoline (N1/C1–C9/Cl2) and 6-chloroquinoline (N2/C11—C19/Cl1) rings are almost planar, with maximal deviations from their mean planes of 0.072 (1) and of 0.044 (1) Å for Cl1 and Cl2 atoms, respectively. The interplanar angle between these rings is 14.36 (5)°. The interplanar angle between the quinoline (N2/C11—C19) and the phenyl (C20–C25) rings equals to 66.00 (8)° while the dihedral angle between the quinoline ring (N2/C11—C19) and the acetaldehyde (C26/C27/O2) group equals to 76.41 (9)°.

The molecules are linked by intermolecular C—H···O interactions (Tab. 1). The crystal structure is further stabilized by C—H···π-electron ring interactions (Tab. 1) and by π-electron···π-electron ring interactions between the pyridine ring (N2/C11– C19; its centroid is Cg1) with each of the benzene rings (C4–C9; its centroid is Cg2) and (C14–C19; its centroid is Cg3). The distances between these centroids of the respective rings are: Cg1···Cg2(1-*x*, 1-*y*, 1-*z*) = 3.7453 (10) Å and Cg1···Cg3 (1-*x*, 1-*y*, 2-*z*) = 3.7557 (9) Å.

### Experimental

To a solution of 3-acetyl-6-chloro-2-hydroxy-4-phenylquinoline (297 mg, 1 mmol) in 5 ml of dimethylsulphoxide) were added solid 2-chloro-3-chloromethylquinoline (211 mg, 1 mmol) and powder Ag_2_SO_4_ (30 mg, 0.1 mmol). Then the mixture was refluxed at 383 K. The reaction was completed in 20 min, having been monitored by the thin layer chromatography using petroleum ether/ethyl acetate (95:5) as an eluant. The reaction mixture was then filtered to remove the catalyst, Ag_2_SO_4_. The filtrate liquid was added dropwise into 50 g of crushed ice. The solution was neutralized by 20 ml of 2N HCl. The precipitate was filtered, dried and re-crystallized from 10 ml of ethanol. The solution was kept for a day after which the resulting crystals were isolated and dried. Colourless block-shaped crystals measured about 0.20 mm in each direction.

### Refinement

All the hydrogens were discernible in the difference electron density maps. However, they were constrained by the riding model approximation: C—H_methylene_=0.97 Å; C—H_methyl_=0.96 Å; C—H_aryl_=0.93 Å; *U*_iso_(H_methylene_/_aryl_)=1.2*U*_eq_(C_methylene_/_aryl_); *U*_iso_H_(methyl_)=1.5*U*_eq_(C_methyl_).

### Crystal data

**Table:**

C_27_H_18_Cl_2_N_2_O_2_	*Z* = 2
*M**_r_* = 473.33	*F*(000) = 488
Triclinic, *P*1	*D*_x_ = 1.388 Mg m^−^^3^
Hall symbol: -P 1	Mo *K*α radiation, λ = 0.71073 Å
*a* = 9.2694 (3) Å	Cell parameters from 1523 reflections
*b* = 10.8862 (4) Å	θ = 1.9–21.4°
*c* = 13.0490 (5) Å	µ = 0.32 mm^−^^1^
α = 100.615 (3)°	*T* = 295 K
β = 103.570 (3)°	Block, colourless
γ = 111.894 (4)°	0.25 × 0.21 × 0.14 mm
*V* = 1132.51 (9) Å^3^	

### Data collection

**Table:**

Oxford Xcalibur Eos (Nova) CCD detector diffractometer	4918 independent reflections
Radiation source: Enhance (Mo) X-ray Source	3250 reflections with *I* > 2σ(*I*)
graphite	*R*_int_ = 0.037
ω scans	θ_max_ = 27.0°, θ_min_ = 3.1°
Absorption correction: multi-scan (*CrysAlis PRO* RED; Oxford Diffraction, 2009)	*h* = −11→11
*T*_min_ = 0.925, *T*_max_ = 0.957	*k* = −13→13
24246 measured reflections	*l* = −16→16

### Refinement

**Table:**

Refinement on *F*^2^	Secondary atom site location: difference Fourier map
Least-squares matrix: full	Hydrogen site location: difference Fourier map
*R*[*F*^2^ > 2σ(*F*^2^)] = 0.040	H-atom parameters constrained
*wR*(*F*^2^) = 0.109	*w* = 1/[σ^2^(*F*_o_^2^) + (0.0549*P*)^2^] where *P* = (*F*_o_^2^ + 2*F*_c_^2^)/3
*S* = 1.05	(Δ/σ)_max_ = 0.001
4918 reflections	Δρ_max_ = 0.27 e Å^−^^3^
300 parameters	Δρ_min_ = −0.35 e Å^−^^3^
0 restraints	Extinction correction: *SHELXL97* (Sheldrick, 2008), FC^*^=KFC[1+0.001XFC^2^Λ^3^/SIN(2Θ)]^-1/4^
71 constraints	Extinction coefficient: 0.0099 (17)
Primary atom site location: structure-invariant direct methods	

### Special details

**Table:**

Geometry. Bond distances, angles etc. have been calculated using the rounded fractional coordinates. All su's are estimated from the variances of the (full) variance-covariance matrix. The cell esds are taken into account in the estimation of distances, angles and torsion angles
Refinement. Refinement on *F*^2^ for ALL reflections except those flagged by the user for potential systematic errors. Weighted *R*-factors wR and all goodnesses of fit *S* are based on *F*^2^, conventional *R*-factors *R* are based on *F*, with *F* set to zero for negative *F*^2^. The observed criterion of *F*^2^ > σ(*F*^2^) is used only for calculating -*R*-factor-obs etc. and is not relevant to the choice of reflections for refinement. *R*-factors based on *F*^2^ are statistically about twice as large as those based on *F*, and *R*-factors based on ALL data will be even larger.

### Fractional atomic coordinates and isotropic or equivalent isotropic displacement parameters (Å^2^)

**Table:**

	*x*	*y*	*z*	*U*_iso_*/*U*_eq_	
Cl1	−0.01220 (6)	0.20006 (6)	1.04669 (4)	0.0754 (2)	
Cl2	0.74140 (7)	0.93000 (5)	0.71360 (5)	0.0840 (2)	
O1	0.59905 (14)	0.49825 (11)	0.72135 (10)	0.0560 (4)	
O2	0.74428 (17)	0.29669 (16)	0.77583 (12)	0.0814 (6)	
N1	0.84627 (17)	0.84682 (14)	0.56079 (12)	0.0521 (5)	
N2	0.43430 (15)	0.49906 (13)	0.82893 (10)	0.0430 (4)	
C1	0.7687 (2)	0.80443 (17)	0.62730 (14)	0.0484 (6)	
C2	0.70647 (18)	0.66777 (16)	0.63617 (13)	0.0432 (5)	
C3	0.73284 (19)	0.57472 (16)	0.56702 (13)	0.0453 (5)	
C4	0.8507 (2)	0.52185 (18)	0.42066 (13)	0.0520 (6)	
C5	0.9361 (2)	0.56806 (19)	0.35310 (14)	0.0564 (6)	
C6	0.9910 (2)	0.7072 (2)	0.35350 (15)	0.0575 (7)	
C7	0.9603 (2)	0.79738 (19)	0.42130 (14)	0.0560 (6)	
C8	0.87261 (19)	0.75263 (16)	0.49223 (13)	0.0445 (5)	
C9	0.81637 (19)	0.61318 (16)	0.49267 (13)	0.0428 (5)	
C10	0.6179 (2)	0.63335 (16)	0.71731 (14)	0.0486 (6)	
C11	0.50542 (19)	0.43665 (16)	0.77951 (13)	0.0431 (5)	
C12	0.49480 (18)	0.30284 (15)	0.77809 (12)	0.0416 (5)	
C13	0.39256 (18)	0.22631 (15)	0.82609 (12)	0.0388 (5)	
C14	0.19783 (18)	0.21883 (17)	0.93163 (13)	0.0459 (5)	
C15	0.12456 (19)	0.28562 (18)	0.98332 (14)	0.0495 (6)	
C16	0.1567 (2)	0.42295 (19)	0.98914 (14)	0.0530 (6)	
C17	0.25945 (19)	0.49098 (17)	0.93803 (13)	0.0485 (6)	
C18	0.33581 (18)	0.42562 (15)	0.88126 (12)	0.0390 (5)	
C19	0.30637 (17)	0.28760 (15)	0.87940 (12)	0.0385 (5)	
C20	0.37473 (19)	0.08368 (15)	0.82266 (13)	0.0416 (5)	
C21	0.2255 (2)	−0.03105 (17)	0.76412 (15)	0.0575 (6)	
C22	0.2112 (3)	−0.16304 (18)	0.75627 (17)	0.0661 (7)	
C23	0.3442 (3)	−0.18296 (19)	0.80755 (16)	0.0639 (8)	
C24	0.4922 (3)	−0.0704 (2)	0.86684 (15)	0.0587 (7)	
C25	0.5077 (2)	0.06250 (17)	0.87376 (13)	0.0481 (6)	
C26	0.6008 (2)	0.25390 (17)	0.72580 (14)	0.0506 (6)	
C27	0.5199 (3)	0.1518 (2)	0.61324 (16)	0.0879 (9)	
H3	0.69470	0.48340	0.56900	0.0540*	
H4	0.81460	0.42950	0.41950	0.0620*	
H5	0.95820	0.50720	0.30630	0.0680*	
H6	1.04900	0.73760	0.30680	0.0690*	
H7	0.99740	0.88930	0.42100	0.0670*	
H10A	0.68150	0.70080	0.78980	0.0580*	
H10B	0.51100	0.63400	0.69340	0.0580*	
H14	0.17640	0.12800	0.93070	0.0550*	
H16	0.10850	0.46760	1.02750	0.0640*	
H17	0.27950	0.58200	0.94070	0.0580*	
H21	0.13420	−0.01860	0.72980	0.0690*	
H22	0.11080	−0.23920	0.71600	0.0790*	
H23	0.33410	−0.27240	0.80220	0.0770*	
H24	0.58240	−0.08340	0.90250	0.0700*	
H25	0.60880	0.13830	0.91330	0.0580*	
H27A	0.59860	0.12490	0.59180	0.1320*	
H27B	0.47890	0.19330	0.56160	0.1320*	
H27C	0.43000	0.07140	0.61380	0.1320*	

### Atomic displacement parameters (Å^2^)

**Table:**

	*U*^11^	*U*^22^	*U*^33^	*U*^12^	*U*^13^	*U*^23^
Cl1	0.0653 (3)	0.0939 (4)	0.0895 (4)	0.0336 (3)	0.0538 (3)	0.0437 (3)
Cl2	0.1264 (5)	0.0624 (3)	0.1087 (4)	0.0548 (3)	0.0834 (4)	0.0406 (3)
O1	0.0692 (8)	0.0515 (7)	0.0724 (8)	0.0302 (6)	0.0473 (7)	0.0353 (6)
O2	0.0569 (9)	0.1003 (11)	0.0888 (10)	0.0354 (8)	0.0381 (8)	0.0119 (8)
N1	0.0597 (9)	0.0463 (8)	0.0640 (9)	0.0239 (7)	0.0349 (8)	0.0269 (7)
N2	0.0441 (7)	0.0441 (7)	0.0467 (8)	0.0205 (6)	0.0192 (6)	0.0191 (6)
C1	0.0508 (10)	0.0473 (10)	0.0576 (10)	0.0235 (8)	0.0273 (9)	0.0226 (8)
C2	0.0393 (9)	0.0438 (9)	0.0487 (9)	0.0154 (7)	0.0179 (8)	0.0202 (8)
C3	0.0451 (9)	0.0393 (9)	0.0506 (10)	0.0130 (7)	0.0182 (8)	0.0200 (8)
C4	0.0587 (11)	0.0490 (10)	0.0487 (10)	0.0233 (9)	0.0189 (9)	0.0149 (8)
C5	0.0596 (11)	0.0669 (12)	0.0506 (10)	0.0324 (10)	0.0236 (9)	0.0183 (9)
C6	0.0560 (11)	0.0721 (13)	0.0551 (11)	0.0276 (10)	0.0299 (9)	0.0286 (10)
C7	0.0620 (11)	0.0554 (11)	0.0622 (11)	0.0240 (9)	0.0333 (10)	0.0306 (9)
C8	0.0427 (9)	0.0471 (9)	0.0485 (10)	0.0187 (8)	0.0200 (8)	0.0210 (8)
C9	0.0398 (9)	0.0454 (9)	0.0421 (9)	0.0160 (7)	0.0130 (7)	0.0173 (7)
C10	0.0521 (10)	0.0461 (9)	0.0573 (10)	0.0214 (8)	0.0278 (9)	0.0247 (8)
C11	0.0455 (9)	0.0442 (9)	0.0465 (9)	0.0181 (8)	0.0242 (8)	0.0205 (7)
C12	0.0438 (9)	0.0427 (9)	0.0432 (9)	0.0199 (7)	0.0200 (8)	0.0146 (7)
C13	0.0396 (8)	0.0387 (8)	0.0385 (8)	0.0159 (7)	0.0151 (7)	0.0122 (7)
C14	0.0429 (9)	0.0471 (9)	0.0511 (10)	0.0171 (8)	0.0210 (8)	0.0209 (8)
C15	0.0413 (9)	0.0642 (11)	0.0512 (10)	0.0229 (8)	0.0248 (8)	0.0240 (9)
C16	0.0511 (10)	0.0722 (12)	0.0533 (10)	0.0377 (9)	0.0273 (9)	0.0218 (9)
C17	0.0523 (10)	0.0517 (10)	0.0517 (10)	0.0297 (9)	0.0207 (9)	0.0189 (8)
C18	0.0372 (8)	0.0431 (9)	0.0385 (8)	0.0177 (7)	0.0139 (7)	0.0144 (7)
C19	0.0353 (8)	0.0423 (9)	0.0387 (8)	0.0158 (7)	0.0140 (7)	0.0139 (7)
C20	0.0476 (9)	0.0400 (9)	0.0433 (9)	0.0183 (8)	0.0248 (8)	0.0153 (7)
C21	0.0534 (11)	0.0467 (10)	0.0685 (12)	0.0185 (9)	0.0195 (9)	0.0165 (9)
C22	0.0716 (13)	0.0425 (10)	0.0789 (14)	0.0162 (10)	0.0322 (11)	0.0158 (10)
C23	0.0994 (16)	0.0485 (11)	0.0689 (12)	0.0401 (12)	0.0496 (12)	0.0287 (10)
C24	0.0805 (14)	0.0709 (13)	0.0551 (11)	0.0502 (12)	0.0367 (11)	0.0312 (10)
C25	0.0533 (10)	0.0525 (10)	0.0452 (9)	0.0253 (8)	0.0228 (8)	0.0164 (8)
C26	0.0604 (11)	0.0532 (10)	0.0562 (11)	0.0298 (9)	0.0356 (10)	0.0249 (9)
C27	0.1010 (17)	0.1078 (18)	0.0619 (13)	0.0604 (15)	0.0312 (13)	0.0040 (12)

### Geometric parameters (Å, °)

**Table:**

Cl1—C15	1.747 (2)	C17—C18	1.408 (3)
Cl2—C1	1.7394 (19)	C18—C19	1.418 (2)
O1—C10	1.427 (2)	C20—C21	1.386 (3)
O1—C11	1.357 (2)	C20—C25	1.378 (3)
O2—C26	1.196 (3)	C21—C22	1.375 (3)
N1—C1	1.295 (2)	C22—C23	1.371 (4)
N1—C8	1.365 (2)	C23—C24	1.372 (3)
N2—C11	1.298 (2)	C24—C25	1.383 (3)
N2—C18	1.374 (2)	C26—C27	1.488 (3)
C1—C2	1.419 (2)	C3—H3	0.9300
C2—C3	1.361 (2)	C4—H4	0.9300
C2—C10	1.503 (2)	C5—H5	0.9300
C3—C9	1.406 (2)	C6—H6	0.9300
C4—C5	1.360 (3)	C7—H7	0.9300
C4—C9	1.416 (3)	C10—H10A	0.9700
C5—C6	1.405 (3)	C10—H10B	0.9700
C6—C7	1.354 (3)	C14—H14	0.9300
C7—C8	1.407 (3)	C16—H16	0.9300
C8—C9	1.411 (2)	C17—H17	0.9300
C11—C12	1.419 (2)	C21—H21	0.9300
C12—C13	1.366 (2)	C22—H22	0.9300
C12—C26	1.512 (3)	C23—H23	0.9300
C13—C19	1.433 (2)	C24—H24	0.9300
C13—C20	1.490 (2)	C25—H25	0.9300
C14—C15	1.359 (3)	C27—H27A	0.9600
C14—C19	1.409 (2)	C27—H27B	0.9600
C15—C16	1.395 (3)	C27—H27C	0.9600
C16—C17	1.362 (3)		
			
Cl1···C22^i^	3.497 (3)	C26···C25	3.120 (2)
Cl2···C24^ii^	3.391 (3)	C27···C20	3.400 (3)
Cl1···H10A^iii^	2.9500	C1···H27B^v^	2.9600
Cl2···H10A	2.7700	C1···H21^vi^	3.0100
Cl2···H10B	3.0500	C4···H10B^v^	2.9700
O1···O2	3.076 (2)	C4···H4^iv^	3.1000
O2···O1	3.076 (2)	C12···H25	3.0200
O2···C25	3.360 (2)	C14···H21	3.1000
O2···C6^iv^	3.296 (3)	C15···H10A^iii^	3.0300
O1···H3	2.3600	C16···H16^x^	3.0900
O2···H25	2.8900	C20···H14	2.6900
O2···H6^iv^	2.5200	C20···H27C	2.8800
O2···H16^iii^	2.8900	C21···H14	2.7600
N2···C5^v^	3.410 (2)	C26···H25	2.9500
N1···H21^vi^	2.7000	H3···O1	2.3600
N1···H7^vii^	2.6300	H3···H4	2.5400
N1···H27B^v^	2.8900	H4···H3	2.5400
N2···H10A	2.7500	H4···C4^iv^	3.1000
N2···H10B	2.5600	H6···O2^iv^	2.5200
C4···C4^iv^	3.291 (3)	H7···N1^vii^	2.6300
C5···C18^v^	3.507 (2)	H10A···Cl2	2.7700
C5···N2^v^	3.410 (2)	H10A···N2	2.7500
C6···C18^v^	3.376 (2)	H10A···Cl1^iii^	2.9500
C6···O2^iv^	3.296 (3)	H10A···C15^iii^	3.0300
C11···C17^iii^	3.583 (2)	H10B···Cl2	3.0500
C11···C16^iii^	3.399 (2)	H10B···N2	2.5600
C14···C21	3.292 (3)	H10B···C4^v^	2.9700
C16···C11^iii^	3.399 (2)	H14···C20	2.6900
C17···C11^iii^	3.583 (2)	H14···C21	2.7600
C18···C5^v^	3.507 (2)	H16···O2^iii^	2.8900
C18···C6^v^	3.376 (2)	H16···C16^x^	3.0900
C18···C18^iii^	3.397 (2)	H16···H16^x^	2.3700
C20···C27	3.400 (3)	H21···N1^xi^	2.7000
C21···C14	3.292 (3)	H21···C1^xi^	3.0100
C22···Cl1^i^	3.497 (3)	H21···C14	3.1000
C24···C24^viii^	3.476 (3)	H25···O2	2.8900
C24···C25^viii^	3.370 (3)	H25···C12	3.0200
C24···Cl2^ix^	3.391 (3)	H25···C26	2.9500
C25···O2	3.360 (2)	H27B···N1^v^	2.8900
C25···C24^viii^	3.370 (3)	H27B···C1^v^	2.9600
C25···C26	3.120 (2)	H27C···C20	2.8800
			
C10—O1—C11	118.10 (14)	C20—C21—C22	120.62 (19)
C1—N1—C8	117.82 (15)	C21—C22—C23	120.4 (2)
C11—N2—C18	116.19 (14)	C22—C23—C24	119.7 (2)
Cl2—C1—N1	115.52 (14)	C23—C24—C25	120.1 (2)
Cl2—C1—C2	118.16 (14)	C20—C25—C24	120.63 (18)
N1—C1—C2	126.32 (17)	O2—C26—C12	119.55 (16)
C1—C2—C3	115.29 (16)	O2—C26—C27	122.6 (2)
C1—C2—C10	120.45 (15)	C12—C26—C27	117.83 (18)
C3—C2—C10	124.27 (15)	C2—C3—H3	119.00
C2—C3—C9	121.62 (16)	C9—C3—H3	119.00
C5—C4—C9	120.58 (17)	C5—C4—H4	120.00
C4—C5—C6	120.40 (18)	C9—C4—H4	120.00
C5—C6—C7	120.58 (18)	C4—C5—H5	120.00
C6—C7—C8	120.27 (18)	C6—C5—H5	120.00
N1—C8—C7	118.72 (16)	C5—C6—H6	120.00
N1—C8—C9	121.43 (16)	C7—C6—H6	120.00
C7—C8—C9	119.84 (16)	C6—C7—H7	120.00
C3—C9—C4	124.14 (16)	C8—C7—H7	120.00
C3—C9—C8	117.52 (16)	O1—C10—H10A	110.00
C4—C9—C8	118.32 (16)	O1—C10—H10B	110.00
O1—C10—C2	106.79 (14)	C2—C10—H10A	110.00
O1—C11—N2	120.52 (15)	C2—C10—H10B	110.00
O1—C11—C12	113.48 (15)	H10A—C10—H10B	109.00
N2—C11—C12	126.00 (16)	C15—C14—H14	120.00
C11—C12—C13	118.51 (16)	C19—C14—H14	120.00
C11—C12—C26	118.07 (15)	C15—C16—H16	120.00
C13—C12—C26	123.40 (15)	C17—C16—H16	120.00
C12—C13—C19	118.23 (15)	C16—C17—H17	119.00
C12—C13—C20	119.71 (16)	C18—C17—H17	120.00
C19—C13—C20	122.06 (15)	C20—C21—H21	120.00
C15—C14—C19	119.79 (16)	C22—C21—H21	120.00
Cl1—C15—C14	119.98 (15)	C21—C22—H22	120.00
Cl1—C15—C16	118.08 (15)	C23—C22—H22	120.00
C14—C15—C16	121.94 (18)	C22—C23—H23	120.00
C15—C16—C17	119.32 (18)	C24—C23—H23	120.00
C16—C17—C18	121.02 (17)	C23—C24—H24	120.00
N2—C18—C17	117.92 (15)	C25—C24—H24	120.00
N2—C18—C19	123.13 (15)	C20—C25—H25	120.00
C17—C18—C19	118.95 (15)	C24—C25—H25	120.00
C13—C19—C14	123.33 (15)	C26—C27—H27A	109.00
C13—C19—C18	117.73 (15)	C26—C27—H27B	109.00
C14—C19—C18	118.92 (15)	C26—C27—H27C	109.00
C13—C20—C21	120.60 (16)	H27A—C27—H27B	110.00
C13—C20—C25	120.80 (15)	H27A—C27—H27C	109.00
C21—C20—C25	118.55 (16)	H27B—C27—H27C	109.00
			
C10—O1—C11—C12	179.97 (14)	C11—C12—C13—C20	178.02 (14)
C10—O1—C11—N2	0.6 (2)	C26—C12—C13—C19	175.94 (15)
C11—O1—C10—C2	−172.51 (14)	C26—C12—C13—C20	−3.5 (2)
C1—N1—C8—C7	−178.41 (17)	C11—C12—C26—O2	77.7 (2)
C1—N1—C8—C9	0.5 (3)	C11—C12—C26—C27	−103.07 (19)
C8—N1—C1—C2	−0.3 (3)	C13—C12—C26—O2	−100.8 (2)
C8—N1—C1—Cl2	179.24 (13)	C13—C12—C26—C27	78.5 (2)
C18—N2—C11—O1	177.01 (14)	C12—C13—C19—C14	180.00 (15)
C18—N2—C11—C12	−2.3 (2)	C12—C13—C19—C18	−1.5 (2)
C11—N2—C18—C17	178.56 (15)	C20—C13—C19—C14	−0.5 (2)
C11—N2—C18—C19	−2.3 (2)	C20—C13—C19—C18	177.98 (14)
N1—C1—C2—C3	0.0 (3)	C12—C13—C20—C21	−114.4 (2)
Cl2—C1—C2—C3	−179.53 (14)	C12—C13—C20—C25	63.0 (2)
Cl2—C1—C2—C10	0.7 (2)	C19—C13—C20—C21	66.2 (2)
N1—C1—C2—C10	−179.77 (18)	C19—C13—C20—C25	−116.50 (19)
C1—C2—C3—C9	0.1 (3)	C19—C14—C15—Cl1	179.18 (13)
C10—C2—C3—C9	179.87 (17)	C19—C14—C15—C16	−1.8 (3)
C3—C2—C10—O1	11.0 (2)	C15—C14—C19—C13	178.00 (16)
C1—C2—C10—O1	−169.28 (15)	C15—C14—C19—C18	−0.5 (2)
C2—C3—C9—C4	178.49 (17)	Cl1—C15—C16—C17	−178.30 (14)
C2—C3—C9—C8	0.1 (3)	C14—C15—C16—C17	2.6 (3)
C9—C4—C5—C6	−0.3 (3)	C15—C16—C17—C18	−1.2 (3)
C5—C4—C9—C3	−178.26 (18)	C16—C17—C18—N2	178.19 (16)
C5—C4—C9—C8	0.1 (3)	C16—C17—C18—C19	−1.0 (2)
C4—C5—C6—C7	0.2 (3)	N2—C18—C19—C13	4.1 (2)
C5—C6—C7—C8	−0.1 (3)	N2—C18—C19—C14	−177.32 (15)
C6—C7—C8—C9	0.0 (3)	C17—C18—C19—C13	−176.73 (15)
C6—C7—C8—N1	178.91 (17)	C17—C18—C19—C14	1.9 (2)
N1—C8—C9—C3	−0.4 (3)	C13—C20—C21—C22	176.68 (18)
N1—C8—C9—C4	−178.90 (16)	C25—C20—C21—C22	−0.7 (3)
C7—C8—C9—C3	178.51 (17)	C13—C20—C25—C24	−177.52 (17)
C7—C8—C9—C4	0.0 (3)	C21—C20—C25—C24	−0.1 (3)
O1—C11—C12—C13	−174.57 (14)	C20—C21—C22—C23	0.8 (3)
O1—C11—C12—C26	6.9 (2)	C21—C22—C23—C24	−0.1 (3)
N2—C11—C12—C13	4.7 (3)	C22—C23—C24—C25	−0.8 (3)
N2—C11—C12—C26	−173.79 (16)	C23—C24—C25—C20	0.9 (3)
C11—C12—C13—C19	−2.5 (2)		

Symmetry codes: (i) −*x*, −*y*, −*z*+2; (ii) *x*, *y*+1, *z*; (iii) −*x*+1, −*y*+1, −*z*+2; (iv) −*x*+2, −*y*+1, −*z*+1; (v) −*x*+1, −*y*+1, −*z*+1; (vi) *x*+1, *y*+1, *z*; (vii) −*x*+2, −*y*+2, −*z*+1; (viii) −*x*+1, −*y*, −*z*+2; (ix) *x*, *y*−1, *z*; (x) −*x*, −*y*+1, −*z*+2; (xi) *x*−1, *y*−1, *z*.

### Hydrogen-bond geometry (Å, °)

**Table:**

Cg1 is the centroid of the N1/C1–C3/C8/C9 ring.

**Table:**

*D*—H···*A*	*D*—H	H···*A*	*D*···*A*	*D*—H···*A*
C3—H3···O1	0.93	2.36	2.703 (2)	101
C6—H6···O2^iv^	0.93	2.52	3.296 (3)	142
C22—H22···Cg1^xi^	0.93	2.95	3.683 (3)	137

Symmetry codes: (iv) −*x*+2, −*y*+1, −*z*+1; (xi) *x*−1, *y*−1, *z*.
